# Widen the Applicability of a Convolutional Neural-Network-Assisted Glaucoma Detection Algorithm of Limited Training Images across Different Datasets

**DOI:** 10.3390/biomedicines10061314

**Published:** 2022-06-03

**Authors:** Yu-Chieh Ko, Wei-Shiang Chen, Hung-Hsun Chen, Tsui-Kang Hsu, Ying-Chi Chen, Catherine Jui-Ling Liu, Henry Horng-Shing Lu

**Affiliations:** 1 Department of Ophthalmology, Taipei Veterans General Hospital, 201 Sec. 2, Shihpai Rd., Taipei 11217, Taiwan; yuchiehko61@gmail.com; 2 Faculty of Medicine, National Yang Ming Chiao Tung University School of Medicine, 155 Sec. 2, Linong St., Taipei 11221, Taiwan; 3 Institute of Statistics, National Yang Ming Chiao Tung University, 4F, Assembly Building I, 1001 University Rd., Hsinchu 30010, Taiwan; wschen.sc06@nycu.edu.tw; 4 Program of Artificial intelligence & Information Security, Fu Jen Catholic University, 510, Zhongzheng Rd., New Taipei City 24205, Taiwan; 152228@mail.fju.edu.tw; 5 Department of Ophthalmology, Cheng Hsin General Hospital, 45 Zhenxing St., Taipei 11240, Taiwan; jackym076@gmail.com; 6 College of Engineering, University of Michigan, 2260 Hayward St., Ann Arbor, MI 48109, USA; brianc.boston@gmail.com

**Keywords:** deep learning, diagnosis, fundus photograph, glaucoma

## Abstract

Automated glaucoma detection using deep learning may increase the diagnostic rate of glaucoma to prevent blindness, but generalizable models are currently unavailable despite the use of huge training datasets. This study aims to evaluate the performance of a convolutional neural network (CNN) classifier trained with a limited number of high-quality fundus images in detecting glaucoma and methods to improve its performance across different datasets. A CNN classifier was constructed using EfficientNet B3 and 944 images collected from one medical center (core model) and externally validated using three datasets. The performance of the core model was compared with (1) the integrated model constructed by using all training images from the four datasets and (2) the dataset-specific model built by fine-tuning the core model with training images from the external datasets. The diagnostic accuracy of the core model was 95.62% but dropped to ranges of 52.5–80.0% on the external datasets. Dataset-specific models exhibited superior diagnostic performance on the external datasets compared to other models, with a diagnostic accuracy of 87.50–92.5%. The findings suggest that dataset-specific tuning of the core CNN classifier effectively improves its applicability across different datasets when increasing training images fails to achieve generalization.

## 1. Introduction

Glaucoma is one of the leading causes of irreversible blindness, but under-diagnosed worldwide [1,2,3,4]. With the advancement of image capture technology for fundus photography and artificial-intelligence-assisted image diagnosis [5,6,7], automated glaucoma detection using fundus photographs may be an effective approach to increase the diagnostic rate of glaucoma through clinic- or population-based glaucoma screening [8,9]. This evolution may save the vision of millions of people at risk of glaucoma blindness [1,2].

The thriving development of deep learning (DL) techniques, especially the use of convolutional neural networks (CNNs), has given rise to new possibilities in medical image analysis, especially in the field of retinal disorders [6,10,11]. DL-based screening of diabetic retinopathy (DR) has been validated clinically and commercially incorporated into fundus photography [6,11]. Contrarily, DL-assisted glaucoma detection, although having reached high sensitivity and specificity in detecting glaucomatous optic neuropathy (GON) using fundus images [12,13,14], has not been applied in daily clinic because of inadequate generalizability. Building an automated glaucoma detection algorithm is more challenging than it is for automated DR screening for two reasons. First, the diagnosis of glaucoma is more complicated, necessitating additional structural and functional evaluations [15,16]. Therefore, it is costly and difficult to build large image datasets containing fundus images with comprehensive glaucoma evaluation. Second, detecting glaucoma using fundus images requires more on the details than DR, such as the clarity of the retinal nerve fiber layers (RNFL) and optic nerve head (ONH) [15,16,17,18]. Therefore, the performance of a DL classifier for glaucoma can be affected by image quality, layout, and variations related to ocular characteristics, such as myopia and retinal pigmentation. With the largest number of images to date—241,032 images from 68,013 patients—available to develop a CNN classifier for automated GON detection [14], the CNN classifier achieved a high diagnostic accuracy while predicting internal test images; however, its performance worsened when it was applied to images from different healthcare systems and subjects of different ethnicities and images of variable quality, with the area under the receiver operating characteristic (ROC) curve (AUC) dropping from 0.996 to 0.987, 0.923, and 0.823, respectively (Table S1). Therefore, expanding image datasets is not an effective approach to increase the applicability of a CNN classifier for glaucoma detection.

Since expanding image datasets is not an affordable nor an effective strategy to increase the generalizability of glaucoma detection algorithms, we explored the possibility of constructing a DL-assisted glaucoma detection model that is first trained using a limited number of fundus images and then fine-tuned to accommodate the variability of image characteristics across different healthcare systems. This approach is logical and feasible, but has not been evaluated in the detection of glaucoma. In this study, a CNN classifier was first developed using high-quality fundus images from one medical center, and then fine-tuned using external datasets to improve the applicability of the CNN classifier in different healthcare systems using limited training images from the targeted healthcare systems.

## 2. Materials and Methods

**Figure 1:**
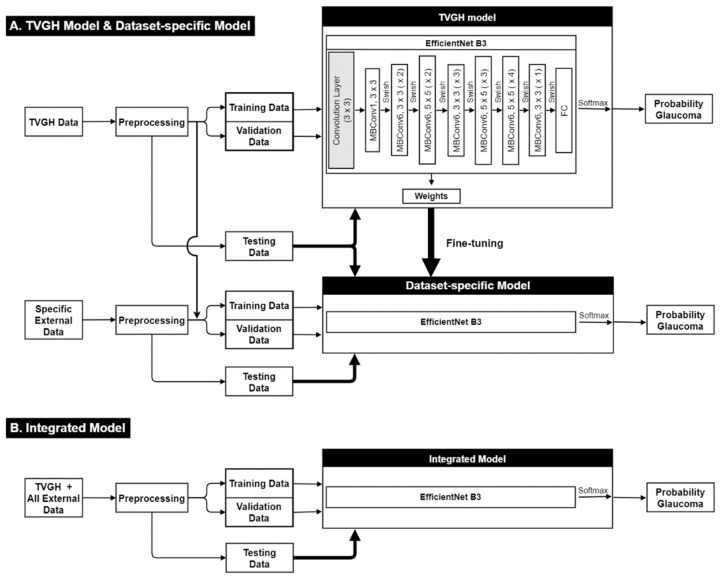
Study framework. The fundus images were first preprocessed with data augmentation before being forwarded to the EfficientNet B3 algorithm to build the deep learning classifier. (**A**) Taipei Veterans General Hospital (TVGH) model was built using images from TVGH dataset and then fine-tuned as dataset-specific model using the weight of the TVGH model and new training images from specific external dataset to improve the diagnostic performance on the specific dataset. (**B**) Integrated model was built using combined images from TVGH dataset and all the external datasets.

The workflow is illustrated in Figure 1. In the first phase of this study, a transfer learning model based on the EfficientNet B3 (https://keras.io/api/applications/efficientnet/, accessed on 16 April 2022) architecture was used to build a CNN classifier using training images of Taipei Veterans General Hospital (TVGH) [19,20], called the ***TVGH model***. The diagnostic accuracies of the TVGH model were evaluated with the TVGH test images and test images of the three external datasets, including two open-access datasets (DRISHTI-GS1 and RIM-ONE r2) and one private dataset (the Cheng Hsin General Hospital (CHGH) dataset) [21,22].

In the second phase of this research, we compared the efficacy of two approaches, the ***dataset-specific model* and the *integrated model*** in increasing the performance of the TVGH model on external datasets, which were too small to train a CNN classifier.

### 2.1. Datasets

The TVGH dataset included fundus photographs, in JPEG format, of 465 non-glaucomatous eyes and 479 eyes with primary open-angle glaucoma (POAG) from the image database of the Department of Ophthalmology of TVGH. Eyes with POAG were diagnosed based on characteristic changes in the ONH and associated reproducible visual field (VF) defects in the presence of a normal open angle on gonioscopy. Glaucomatous ONH changes were defined as an enlarged cup-to-disc ratio along with generalized neuroretinal rim thinning or focal notching. All subjects with POAG underwent VF analysis using the 24-2 Swedish interactive threshold algorithm standard of the Humphrey Field Analyzer 750i (version 4.2, Zeiss-Humphrey Instruments, Dublin, CA, USA) and RNFL scanning using Cirrus high-definition optical coherence tomography (Model 4000; CarlZeiss Meditec, Inc., Dublin, CA, USA). The non-glaucomatous eyes had an intraocular pressure of less than 21 mmHg and an ONH of normal appearance without RNFL defects. 

The image characteristics of the TVGH and external datasets are listed in Table 1. The ground truth for classification was made based on the majority opinion of experts in DRISHTI-GS1 and RIN-ONE r2 datasets, and the consensus of two experts after reviewing detailed clinical information in CHGH dataset (YK and TH) and TVGH dataset (YK and CL). Different from other datasets, the images of RIM-ONE r2 were pre-cropped and centered at the ONH as the region of interest [22].

The images from each dataset were divided into training, validation, and testing images at a sampling ratio of 4:1:1.

### 2.2. Image Classification Based on CNN and Fine-Tuning Process

Fine-tuning is the core of transfer learning, which can be achieved in different schemes regarding the setting of initial weights [23,24]. The first scheme is to retrain the model with new datasets but keeping the weights of the pretrained model as the initial weights; the second is to retrain the model after resetting parts of the weights; and the third is to do so after clearing the weights of the pretrained model. The TVGH model was built following the third scheme, in which the pretrained weights of EfficientNet B3 were discarded and the classifier was retrained by the training images of the TVGH dataset. Thereafter, the TVGH model was fine-tuned according to the first scheme as the dataset-specific models, in which the architecture and initial weights of the TVGH model were kept and fine-tuned using the merged dataset containing the TVGH training set and one of the three external datasets (Figure 1A), specified as DRISHTI-GS1-specific model, RIM-ONE r2-specific model, and CHGH-specific model, respectively. This approach extended the feature learning from the TVGH model to accelerate the convergence of the specific model. Finally, the integrated model was built using the third scheme by retraining the TVGH model with the merged dataset containing training images from the 4 datasets (Figure 1B). This approach aimed to verify whether training set expansion using small external datasets is useful for improving the generalizability of the classifier.

**Figure 2:**
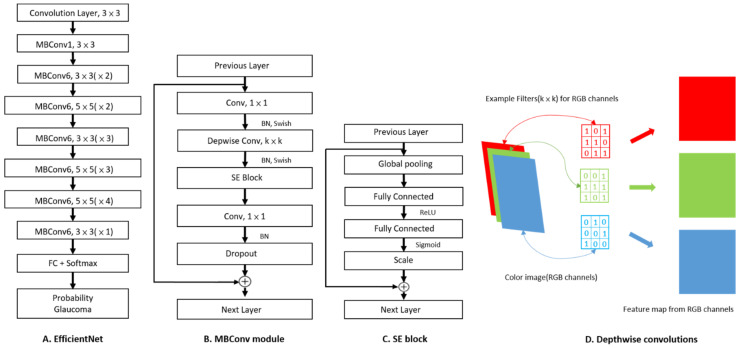
Model and module architecture diagram. (**A**) The architecture of EfficientNet B3, consisting of one convolutional layer and seven MBConv modules. Each MBConv module is followed by a number 1 or 6, which is the multiplication factor *n*. The number *n* means the first 1 × 1 convolutional layer expands the channels by *n* times. (**B**) The architecture of MBConv module. The k × k following Depwise Conv is the kernel size of Depwise Conv in the MBConv module, and listed in (**A**) as 3 × 3 or 5 × 5. (**C**) The architecture of squeeze-and-excitation (SE) block. SE block increases the weight of essential features and reduces the weight of useless features according to the change of loss in the training process to improve the prediction performance. (**D**) Schematic diagram of Depthwise (Depwise) convolutions. Depthwise Conv performs convolution with different filters for each image channel to reduce the computation loading. FC: fully connected layer.

EfficientNet was chosen in this study because it used the Neural Architecture Search [19,20,25] technology for optimization of the number of neurons in the neural network, the depth of the neural network, and the image resolution. Considering the limitation in computing resources, EfficientNet B3 was selected, which consists of one convolutional layer and seven MBConv modules, followed by the fully connected layer (Figure 2). The MBConv module combines residual block, squeeze-and-excitation block, and depthwise convolutions to reduce computational load and improve accuracy [26,27,28]. In this study, the architecture of EfficientNet B3 was maintained, except for replacing the fully connected layer with the one including two neurons only. Softmax function was used as the activation function for binary output.

The color fundus images were first preprocessed to crop the region of interest as a square. As the default resolution of the input images to the EfficientNet B3 is 300 × 300, we resized the images to match this specification by importing the Python library OpenCV (Figure S1). Each pixel has three channels, red, green, and blue. Data augmentation was performed to improve the model performance and avoid overfitting with the following steps. Images were randomly rotated by 30°, horizontally or vertically flipped, or shifted by 10% of the total width or height.

This work was performed based on an open-source DL framework, TensorFlow (Version 1.13.1, https://github.com/tensorflow/docs/tree/r1.13/site/en/api_docs, accessed on 16 April 2022), and Keras (Version 2.2.4, https://keras.io/api/, accessed on 16 April 2022). An NVIDIA GeForce GTX 1070 Ti GPU (NVIDIA Corporation, Santa Clara, CA, USA) was used. The training dataset was divided into five nonoverlapping folds during the training process. Each of the 5-fold was used as a validation dataset, in turn, to select hyper-parameters and estimate the model’s performance to avoid overfitting. The detailed hyper-parameters of the networks in the different models are listed in Table 2, which were adjusted to make sure the loss of the training and validation set had a similar downward trend. One epoch is when an entire training dataset propagates forward and backward through the algorithm once. However, with the limitation of computing power, the whole training images cannot complete one epoch at once, and are therefore divided into batches, with the number of images used in each batch as batch size. Batch size is adjusted according to image characteristics to achieve convergence and acceptable performance. The number of epochs is determined as the point when there was a smallest difference of loss between the training and the validation set. Learning rate defines the adjustment in the weights of the network with respect to the loss gradient, which affects the speed and convergence of the training process. We set the initial learning rate from 6 × 10^−5^ and adjusted it according to the loss of validation set.

Gradient-weighted class activation mapping (Grad-CAM) was applied to identify features recognized by the proposed CNN classifier. The gradient information from the last convolutional layer in the CNN classifier determined the importance of each filter. Grad-CAM represented the area that the model considered essential by combining all the feature maps from the filters, which was projected back onto the input fundus image to highlight areas critical to classification [29].

### 2.3. Statistical Analysis

The performance of the CNN classifiers was evaluated along the indices of sensitivity, specificity, and accuracy. The ROC curve was plotted using the matplotlib package in Python. A nonparametric test, the Mann—Whitney U test, was used to compare the clinical characteristics of correctly and incorrectly predicted images, because most measurements of the test images were not normally distributed. The analysis was conducted using SPSS (version 18.0.0, SPSS Inc., Chicago, IL, USA).

## 3. Results

### 3.1. Diagnostic Performance of the CNN Classifiers

**Figure 3:**
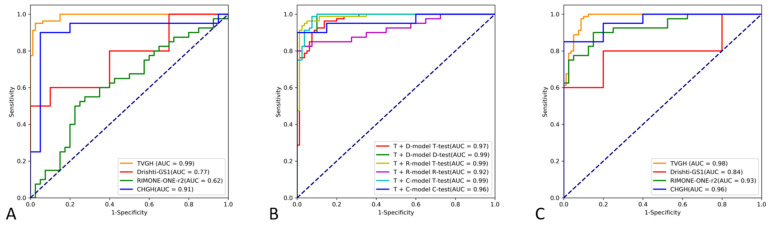
Receiver operating characteristic (ROC) curves of the TVGH model, dataset-specific models, and integrated model to differentiate between normal and glaucomatous fundus images on different test datasets. (**A**) The TVGH model is a CNN classifier constructed with the training images of the TVGH dataset. Different colored lines indicate the results obtained upon using the TVGH model to classify the test images of the TVGH, DRISHTI-GS1, RIM ONE r2, and CHGH datasets. (**B**) ROC curves of the dataset-specific models in predicting test images from the corresponding datasets. The line corresponding to the T + D model D test refers to the predictive result of DRISHTI-GS1-specific model trained with mixed training images from the TVGH and DRISHITI-GS1 datasets on DRISHTI-GS1 test images. T: TVGH; D: DRISHTI-GS1; R: RIM-ONE r2; C: CHGH. (**C**) ROC curves of the integrated model constructed with combined training images from all datasets in detecting glaucoma using various test datasets.

The TVGH model had a diagnostic accuracy of 95.62% on TVGH test images, with a sensitivity, specificity, and AUC of 93.75%, 97.50%, and 0.991, respectively (Table 2). However, the diagnostic accuracy of the TVGH model on DRISHTI-GS1, RIM-ONE r2, and CHGH test images dropped to 55.0%, 52.50%, and 80.00%, with AUCs of 0.770, 0.624, and 0.910, respectively (Table 3 and Figure 3A).

The dataset-specific models, fine-tuned with one specific external dataset had significantly improved diagnostic accuracies on the corresponding test datasets (Table 3 and Figure 3B). For example, the DRISHTI-GS1-specific model had diagnostic AUCs of 0.969 and 0.990 on the TVGH and DRISHTI-GS1 test images, respectively, compared to AUCs of 0.991 and 0.770 when the TVGH model was applied on the TVGH and DRISHTI-GS1 test images, respectively.

The integrated model, constructed by training images from the four datasets, had a diagnostic accuracy on TVGH, DRISHTI-GS1, RIM-ONE r2, and CHGH test images of 91.88%, 50.0%, 82.50%, and 85.0%, with AUCs of 0.981, 0.840, 0.930 and 0.960, respectively (Table 3 and Figure 3C).

Figure S2 revealed the training curves of the TVGH, integrated, and dataset-specific models. The loss of the training and validation datasets decreased with each epoch, without a gap between the two datasets, indicating that the models were stable without overfitting.

**Figure 4:**
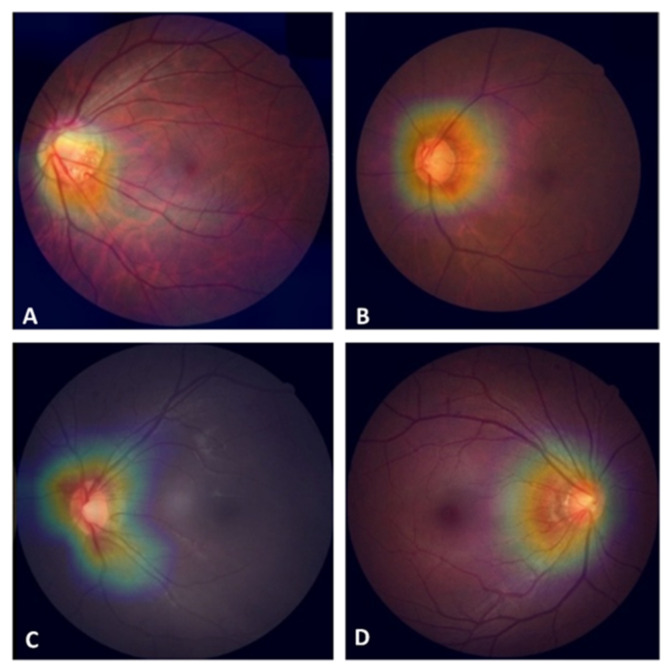
Gradient-weighted class activation mapping (Grad-CAM) identifying features extracted for classification in the Taipei Veterans General Hospital (TVGH) model. The hot spots (red color) were localized at the optic nerve head, with some extending to the peripapillary nerve fiber bundles in both glaucoma (**A**,**B**) and healthy eyes (**C**,**D**).

Grad-CAM revealed that features critical for classification in the TVGH model, marked in red, were located primarily at the ONH and peripapillary nerve fiber bundles (Figure 4).

### 3.2. Factors Affecting the Detection of POAG with the CNN Classifier

To understand whether disease severity affects the performance of the CNN classifier in detecting GON, we compared the clinical and demographic characteristics of the TVGH test images with correct and incorrect classifications of GON (Table 4). There were only two control images misclassified as glaucoma with cup-to-disc ratios of 0.4 and 0.6. The GON images misclassified as normal were from patients with a less severe disease, with an average mean deviation (MD) of −2.43 dB and RNFL thickness of 85.40 μm compared to values of −5.62 dB and 71.96 μm for correctly classified images, respectively. Moreover, all the images misclassified as normal had an early disease with an MD better than −6 dB, ranging from −5.34 to −0.6 dB. 

## 4. Discussion

In this study, the CNN classifier trained with a single dataset (TVGH model) had a diagnostic AUC of 0.991 on test images of the same origin, which is much higher than the AUCs of 0.624 to 0.910 on test images of different origins. Both the integrated model and dataset-specific models had improved diagnostic performance on an external dataset than the TVGH model. The dataset-specific model, a fine-tuned TVGH model by including training images from the targeted external dataset had significantly improved diagnostic accuracy on the specific dataset. The integrated model, which was constructed by combined training images from all different datasets, did not achieve equal performance on each external dataset. The diagnostic AUC of our dataset-specific models on local independent validation dataset, external clinic-based dataset, and public/population datasets of different ethnicity were 0.991, 0.963, and 0.969–0.986, respectively, which were compatible with the AUC values of 0.996, 0.987–0.995, and 0.923–0.964, respectively, in Liu et al.’s DL algorithm trained with 274,413 images. In a recently published meta-analysis including 180,534 fundus images of 67 studies, the pooled mean sensitivity, specificity, and AUC of the glaucoma detection neural networks were 91%, 91%, and 0.96, respectively [30] (Table S1). These findings suggested that, even with limited training images, specifically fine-tuning a core model to accommodate potential variations in the targeted testing population may be an effective approach to increase the applicability of the DL model to assist glaucoma detection. 

Generalizability is a critical issue for the further application of DL-assisted detection of disease using images [31], especially in the detection of GON. We used an ensemble approach by incorporating a support vector machine to identify discs with enlarged cup-to-disc ratio when the confidence score of the CNN classifier was less than 0.85. However, this approach is still limited by the overlapped distribution of cup-to-disc ratio among the healthy and glaucoma subjects [32]. Increasing the number and variability of the training images is a potential way to increase the generalizability of a DL model, but its effect reached plateau when using 60,000 or more training images in the DR model, and is suboptimal in glaucoma models [14,33]. Furthermore, this approach is often not accessible for most researchers and may not be cost-effective when the classifier is designed for glaucoma screening on targeted populations of the same ethnicity or images of controllable layout and quality. Several studies have demonstrated the feasibility of building a CNN classifier for glaucoma using limited training images, and the importance of including images from the targeted dataset for training to improve the diagnostic accuracy on the target. Gómez-Valverde et al. demonstrated that a CNN classifier constructed using a relatively small number of training images can achieve a diagnostic AUC of 0.94 in identifying GON. The training images comprised 370 glaucomatous and 1364 normal images gathered from three datasets. By evaluating various combinations of the three training datasets, they found that the diagnostic performance of the CNN classifier on mixed testing images improved proportionally with the number of additional datasets added during training [34]. However, the performance of the CNN classifier on the specific dataset was not evaluated. Diaz-Pinto et al. used five public datasets with 1707 images to verify the generalizability of their CNN classifier for GON. The AUC of the CNN classifier achieved a value of 0.9605 when training images from all five datasets were included in building the model, but dropped to the range of 0.8575 to 0.7678 when the training images from the desired test dataset were removed [35] (Table S1). The above approaches, as our integrated model, may not increase the diagnostic performance of the model equally across different datasets when the number of images from specific datasets were limited. Contrarily, our study demonstrated another cost-effective approach by building a core CNN classifier using a limited number of high-quality images to extract critical features for glaucoma detection and then fine-tuning the model to improve its applicability in different datasets, as the data-specific model.

Several studies have proposed different approaches to increase the diagnostic power of DL-assisted glaucoma detection using a limited number of training images, which is a universal predicament encountered by researchers. Gheisari et al. [36] and Xu et al. [37] used a limited number of training images, as with the current study, being 1810, 1882, and 944, respectively. Gheisari et al. used serial fundus images of one subject to increase the diagnostic rate; Xu et al. used fundus images taken from cataract patients (Similar Ophthalmic Database, SOD) to replace the ImageNet dataset to improve general feature extraction in transfer learning and used transfer-induced attention network (TIA-Net) to detect specific features of glaucoma. Although the approach used by Gheisari et al. improved the F score significantly from 79.2% to 96.2%, their approach is of less clinical utility because the value of DL is to assist glaucoma screening, in which it is difficult to have serial fundus images taken as a cohort. While the approach by Xu et al. is sound, it does not lead to significant improvement of the diagnostic accuracy: 85.7% using TIA-Net with SOD, compared with 84.1% using ImageNet dataset with CNN. Furthermore, the approach by Xu et al. did not improve the generalizability because the diagnostic accuracy of TIA-Net with SOD dropped a lot when it was applied to an open-access dataset compared with their own dataset, 76.6% vs. 85.7%, respectively (Table S1). Contrarily, the diagnostic accuracy improved from 52–80% to 87.5–95.0% on the external dataset by adopting the dataset-specific model in this study. Considering further verification and application of our approach, we prepared a website based on the TVGH model of this study for researchers who want to upload their own images for verification, http://140.113.114.104/vght_demo/demo-biomedicines, accessed on 16 April 2022. However, further co-operation may be needed to find-tune the TVGH model with images from the external dataset to improve its performance. On the other hand, federated learning may be an alternative to fine-tuning the TVGH model, considering data protection and privacy [38,39].

The TVGH model had a higher diagnostic accuracy on test images from CHGH than those from DRISHTI-GS1 and RIM-ONE r2. The similarity in image layout between TVGH and CHGH datasets, macula-centered 30° fundus images, may be the explanation. Contrarily, images from DRISHTI-GS1 and RIM-ONE r2 are disc-centered, the latter being cropped with limited information from other parts of the retina [21,22]. However, the dataset-specific model retrained with limited training images from the targeted dataset overcame these differences. Grad-CAM of the TVGH model revealed the features critical for classification centered on the ONH. This may explain why the TVGH model can be effectively fine-tuned to fit the DRISHTI-GS1 and RIM-ONE r2 datasets with images centered on the ONH.

Another advantage of our approach is that we can evaluate the diagnostic accuracy of the CNN classifiers on images of different disease severities by using training fundus images with comprehensive clinical information. This is contrary to the approaches which adopted a large number of fundus images without clinical information and identified GON based solely on ONH and RNFL appearance [13,14]. We found that our model correctly classified all images with moderate to severe disease severity, not only for the TVGH test images, but also in the fine-tuned model applied to CHGH test images (data not shown). The issue of the diagnostic accuracy of the DL algorithm on images with different disease severities has not been thoroughly evaluated because most studies used fundus images without clinical information as their training images [13,14]. Similar to our findings, and as is the case for most diagnostic tools for glaucoma, Christopher et al. revealed that the CNN classifier had a better diagnostic performance in eyes with moderate to severe functional loss than those with mild loss [12].

This study has some limitations. First, the images with poor quality were excluded in the datasets. Therefore, the effect of image quality on the performance of our model was not counted in this study. Second, cases with anomalous or extreme disc appearance, such as extremely tilted discs and retinopathies except for drusen, were excluded from the dataset; hence, our model may not be applicable to such cases. Third, different diagnostic criteria across different datasets may affect the performance of the CNN classifier. In TVGH and CHGH datasets, complete clinical assessments, including VF and optical coherence tomography evaluation, were reviewed to achieve the classification. Nevertheless, in DRISHTI-GS1 and RIN-ONE r2 datasets, it is not clear how the diagnosis was made by the experts and whether VF defects were the prerequisite for the diagnosis of glaucoma. Lastly, our centralized approach needs to obtain external training images to fine-tune the TVGH model, which may encounter critical concerns regarding patient privacy and data protection. The next step of this study would be adopting a framework of federated learning to fine-tune the TVGH model across multiple medical institutions without exchange or centralize datasets [38,39,40]. 

In conclusion, we proposed an effective approach to increase the applicability of a CNN classifier for GON detection. A CNN classifier can be built using a limited number of high-quality fundus images and then fine-tuned using a small number of images from the targeted testing dataset to improve its diagnostic performance on the specific dataset. This approach may be a feasible alternative when a large image database of GON is not accessible, given that a generalizable CNN classifier for GON detection is currently unavailable.

## Figures and Tables

**Table 1:** Characteristics of the datasets used in this Study.

Dataset	Numbers (Glaucoma)	Image Size (Pixels)	Field of View (Center)	Camera	Origin
TVGH	944 (479)	3888 × 2552	30° (macula)	Canon CX-1, CR-2, DGi	Taiwan
CHGH	158 (78)	3216 × 2136	30° (macula)	Topcon TRC-NW8F	Taiwan
DRISHTI-GS1	101 (70)	2047 × 1760	30° (disc)	Zeiss Visucam NM/FA	India
RIM-ONE r2	455 (200)	Not fixed	Cropped (disc)	Nidek AFC-210	Spain

TVGH, Taipei Veterans General Hospital; CHGH, Cheng Hsin General Hospital.

**Table 2:** Network hyper-parameters used in different Models.

Models ^1^	Network Hyper-Parameters		
	Batch Size	Epoch	Initial Learning Rate
TVGH	20	20	0.00006
Integrated	16	25	0.00004
DRISHTI-GS1-specific	16	20	0.00001
RIM-ONE r2-specific	16	20	0.00005
CHGH-specific	20	20	0.00006

^1^ Each model was named after the training dataset(s) used for training. TVGH model is the core model of this study built with the architecture of EfficientNet B3 and TVGH training images. Integrated model was built with the architecture of EfficientNet B3 and the training images from all datasets. Dataset-specific model used specific dataset to fine-tune the TVGH model, for example, DRISHTI-GS1-specific model used training images from DRISHTI-GS1 dataset to fine-tune TVGH model. TVGH, Taipei Veterans General Hospital; CHGH, Cheng Hsin General Hospita.

**Table 3:** Diagnostic performance of the deep learning models built with different training datasets on test images across different datasets.

Models ^1^	Training Datasets	Test Datasets	Accuracy	Specificity	Sensitivity	AUC (95% CI)
TVGH	TVGH	TVGH	95.62%	97.50%	93.75%	0.991 (0.982–1.000)
TVGH	TVGH	DRISHTI-GS1	55.00%	100%	10.00%	0.770 (0.558–0.982)
TVGH	TVGH	RIM-ONE r2	52.50%	90.00%	15.00%	0.624 (0.501–0.748)
TVGH	TVGH	CHGH	80.00%	95.00%	65.00%	0.910 (0.798–1.000)
DRISHTI-GS1-specific	TVGH + DRISHTI-GS1	TVGH	88.75%	92.50%	85.00%	0.969 (0.945–0.993)
DRISHTI-GS1-specific	TVGH + DRISHTI-GS1	DRISHTI-GS1	95.00%	90.00%	100.00%	0.990 (0.958–1.000)
RIM-ONE r2-specific	TVGH + RIM-ONE r2	TVGH	94.38%	98.75%	90.00%	0.986 (0.969–1.000)
RIM-ONE r2-specific	TVGH + RIM-ONE r2	RIM-ONE r2	87.50%	92.50%	82.50%	0.922 (0.859–0.985)
CHGH-specific	TVGH + CHGH	TVGH	92.50%	93.75%	91.25%	0.988 (0.977–1.000)
CHGH-specific	TVGH + CHGH	CHGH	92.50%	95.00%	90.00%	0.963 (0.901–1.000)
Integrated	All	TVGH	91.88%	91.25%	92.50%	0.981 (0.965–0.998)
Integrated	All	DRISHTI-GS1	50.00%	20.00%	80.00%	0.840 (0.651–1.0)
Integrated	All	RIM-ONE r2	82.50%	87.50%	77.50%	0.930 (0.875–0.985)
Integrated	All	CHGH	85.00%	75.00%	95.00%	0.960 (0.906–1.0)

^1^ All the model architectures are adopted from the model architecture of EfficientNet B3. Model weights are different due to the various training data and hyperparameters. Each model was named after the training dataset(s) used for training. TVGH model is the core model of this study built with the architecture of EfficientNet B3 and TVGH training images. Dataset-specific model used specific dataset to fine-tune the TVGH model, for example, DRISHTI-GS1-specific model used training images from DRISHTI-GS1 and TVGH datasets to fine-tune TVGH model. Integrated model was built with the architecture of EfficientNet B3 and the training images from all datasets. TVGH, Taipei Veterans General Hospital; CHGH, Cheng Hsin General Hospital; AUC, area under receiver operating characteristic curve; CI: confidence interval.

**Table 4:** Clinical characteristics and prediction accuracy of the TVGH model in images with primary open angle glaucoma.

CNN Prediction			
	Correct (*n* = 75)	Incorrect (*n* = 5)	*p* Value
Age (years)	58.00 ± 14.71	58.00 ± 19.20	0.91
Cup-to-disc ratio	0.79 ± 0.12	0.72 ± 0.11	0.16
Visual field			
MD (dB)	−5.62 ± 5.26	−2.43 ± 2.04	0.09
PSD (dB)	5.71 ± 3.59	3.73 ± 2.25	0.37
Average RNFL thickness (µm)	71.96 ± 10.86	85.40 ± 13.70	0.04

Values are presented as mean ± SD. TVGH, Taipei Veterans General Hospital; CNN, convolutional neural network; dB, decibel; MD, mean deviation; PSD, pattern standard deviation; RNFL, retinal nerve fiber layer.

## Data Availability

The image datasets cannot be shared publicly because of the regulation of the Ministry of Science and Technology of Taiwan. The images have been uploaded in the National Center for High-performance Computing, the LIONS Data Framework and is available for researchers who meet the criteria for accessing to confidential data. The detailed information can be found at http://omim.nchc.org.tw/LIONSdata/search.php, accessed on 16 April 2022.

## Supplementary Materials

The following are available online at ’https://www.mdpi.com/article/10.3390/biomedicines10061314/s1, Table S1 Performance of deep learning models in detecting glaucoma using large training datasets or specific approaches to improve accuracy. Figure S1. The preprocessing process to crop the region of interest as a square. Figure S2. Training curves of the deep leaning models.
