# Supplementary material for: Widen the Applicability of a Convolutional Neural-Network-Assisted Glaucoma Detection Algorithm of Limited Training Images across Different Datasets
Source: Biomedicines. 2022 Jun 3;10(6):1314. doi: 10.3390/biomedicines10061314 (PMC9219722; doi:10.3390/biomedicines10061314)
Supplement: Supplementary file 1 [file biomedicines-10-01314-s001.zip › biomedicines-1727078-supplementary.pdf]

Supplementary Materials for Widen the Applicability of a Convolutional Neural-Network-Assisted Glaucoma Detection Algorithm of Limited Training Images across Different Datasets

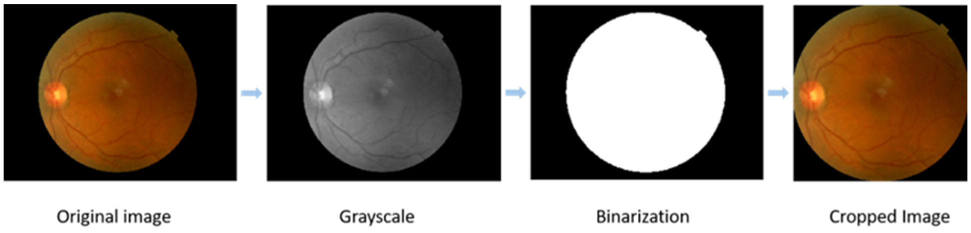

Figure S1. The preprocessing process to crop the region of interest as a square. The fundus images were first converted into grayscale, followed by binarization to crop the image along the boundaries automatically. For images not being square af-ter cropping, the shortage will be filled with zero padding. The cropped images were then re-converted to color images without enhancement of contrast.

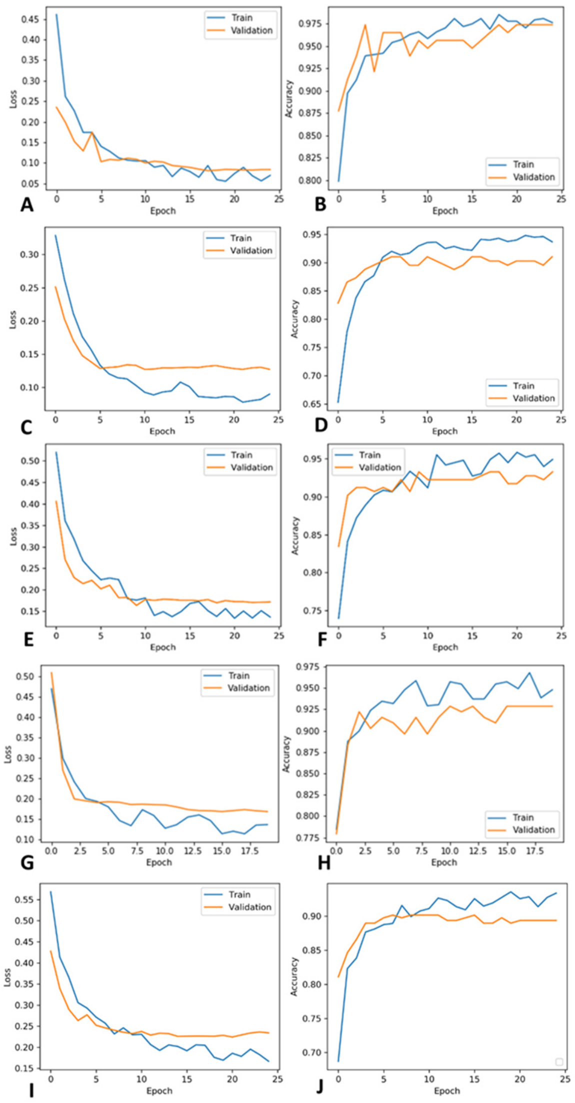

Figure S2. Training curves of the deep leaning models. The blue and orange lines in the left panel (A,C,E,G,I) show that the loss of training and validation datasets decreased over time, respectively; while the accuracy increased over time in the left panel (B,D,F,H,J). With the increase of accuracy, the loss curve of the validation dataset remained stable and approximated that of the training curve indicating that the deep learning models were stable and not overfitted. The loss curves of TVGH (A), DRITSHI-GS1-specific (C), RIN-ONE r2-specific (E), CHGH-specific (G), and integrated (I) models. The accuracy curves of TVGH (B), DRITSHI-GS1-specific (D), RIN-ONE r2-specific (F), CHGH-specific (H), and integrated (J) models.

Table S1. Performance of deep learning models in detecting glaucoma using large training datasets or specific approaches to improve accuracy.

First author, publication year	Classifier platform (Modification)	Image numbers	Validation/testing dataset (Type)	Sensitivity (%)	Specificity (%)	AUC
This study	EfficientNet B3 (Nil)	944	Local independent	93.75	97.50	0.991
	EfficientNet B3 (Integrated)	1658	External clinic	92.50	91.25	0.930
			Public/ different ethnicity	77.5-80.0	20.0-87.5	0.840-0.930
	EfficientNet B3 (Dataset-specific)	944+(158/101/455)	External clinic	90.0	95.0	0.963
			Public/ different ethnicity	82.5-100	90.0-92.5	0.922-0.990
Li, 2018 [13]	Deep learning (Nil)	39745	Local independent	95.6	92.0	0.986
Liu, 2019 [14]	ResNet (Nil)	274413	Local independent	96.2	97.7	0.996
			External clinic	93.6-99.1	95.6-97.1	0.987-0.995
			Population screening	91.0	92.6	0.964
			Multiethnic	87.7	80.8	0.923
			Images of variable quality	82.2	70.4	0.823
Wu, 2022 [30]	Meta-analysis of neural network algorithms	180534	Not specified	0.91	0.91	0.96
Diaz-Pinto, 2019 [35]	Xception(Integrated)	1707 (5 public datasets)	Local independent	0.9346	0.8580	0.9605
		45/101/455/401/705(4 the 5 public dataset)	External (the one not included for training)	68.93-83.33	70.20-79.90	0.7678-0.8575
Gheisari, 2021 [36]	VGG-16+LSTM (Temporal features)	1810(+295 fundus videos)	Local independent	95	96	0.9619
Xu, 2021 [37]	Transfer induced attention network	1882(glaucoma)+10463(cataract)	Local independent	84.9	86.9	0.929
			Public/different ethnicity	75.36	77.2	0.835

AUC: the area under the receiver operating characteristic curve.
